# A Dissociation of the Acute Effects of Bupropion on Positive Emotional Processing and Reward Processing in Healthy Volunteers

**DOI:** 10.3389/fpsyt.2018.00482

**Published:** 2018-10-16

**Authors:** Annabel E. L. Walsh, Nathan T. M. Huneke, Randi Brown, Michael Browning, Phil Cowen, Catherine J. Harmer

**Affiliations:** ^1^NIHR Oxford Health Biomedical Research Centre, Warneford Hospital, Oxford, United Kingdom; ^2^Psychopharmacology and Emotion Research Laboratory, Department of Psychiatry, University of Oxford, Oxford, United Kingdom; ^3^University Department of Psychiatry, University of Southampton, Southampton, United Kingdom; ^4^Oxford Health NHS Foundation Trust, Warneford Hospital, Oxford, United Kingdom

**Keywords:** emotion, antidepressants, dopamine, reward, depression, anhedonia

## Abstract

**Background:** Previous research indicates that antidepressants can restore the balance between negative and positive emotional processing early in treatment, indicating a role of this effect in later mood improvement. However, less is known about the effect of antidepressants on reward processing despite the potential relevance to the treatment of anhedonia. In this study, we investigated the effects of an acute dose of the atypical antidepressant (dual dopamine and noradrenaline reuptake inhibitor) bupropion on behavioral measures of emotional and reward processing in healthy volunteers.

**Methods:** Forty healthy participants were randomly allocated to double-blind intervention with either an acute dose of bupropion or placebo prior to performing the Emotional Test Battery (ETB) and a probabilistic instrumental learning task.

**Results:** Acute bupropion significantly increased the recognition of ambiguous faces as happy, decreased response bias toward sad faces and reduced attentional vigilance for fearful faces compared to placebo. Bupropion also reduced negative bias compared to placebo in the emotional recognition memory task (EMEM). There was no evidence that bupropion enhanced reward processing or learning. Instead, bupropion was associated with reduced likelihood to choose high-probability wins and increased score on a subjective measure of anhedonia.

**Conclusions:** Whilst acute bupropion decreases negative and increases positive emotional processing, it has an adverse effect on reward processing. There seems to be a dissociation of the acute effects of bupropion on positive emotional processing and reward processing, which may have clinical implications for anhedonia early in treatment.

## Introduction

Patients suffering from major depressive disorder (MDD) display negative biases in emotional processing across a range of cognitive domains, including perception, attention, and memory (1–4). The neuropsychological theory of antidepressant action hypothesizes that the direct action of antidepressants is to decrease negative emotional processing and increase positive emotional processing early in treatment, prior to any mood improvement, indicating a role of this change in the therapeutic effect of the antidepressant (4–7). Indeed, acute or 7 day administration of the selective serotonin reuptake inhibitor citalopram or the noradrenaline reuptake inhibitor reboxetine was found to increase the recall of positive self-referent words and the perception of ambiguous faces as happy in both healthy volunteers (8–10) and MDD patients (11) in the absence of any changes in mood.

The majority of research on the effects of antidepressants has been conducted using selective serotonin and/or noradrenaline reuptake inhibitors (SSRIs/SNRIs) and several questions remain. Firstly, MDD is not only characterized by low mood but also a loss of interest or pleasure in previously enjoyed activities, known as anhedonia. It is becoming clearer that whilst SSRIs or SNRIs reduce negative biases in emotional processing to improve low mood, they do not fully correct the experience of anhedonia (12) and may actually exacerbate reward deficits (13). Pre-clinical, physiological studies evidence a role of dopamine in reward (14, 15). Therefore, it has been hypothesized that anhedonia and abnormal reward-based decision making in probabilistic instrumental learning tasks observed in MDD (16–18) involve changes in the dopamine system. Indeed, an acute dose of a dopaminergic enhancing drug (L-DOPA) has previously been found to increase the likelihood of choosing high-probability wins during a probabilistic instrumental learning task compared to a dopamine antagonist (haloperidol) in healthy volunteers (19). It has therefore been suggested that atypical, dopaminergic antidepressants may act on such aberrant reward processing and be better suited to treat anhedonia (12).

It is unclear whether positive emotional processing and reward processing are different expressions of the same underlying system (20), or whether they are independent processes in the manifestation of the symptom clusters in MDD. As such, emotional and reward processing may be either similarly or differentially affected by antidepressants with an effect on dopamine function such as bupropion, a dual dopamine and noradrenaline reuptake inhibitor.

Therefore, here we investigated the acute effects of bupropion compared to placebo on commonly used behavioral measures of emotional and reward processing in healthy volunteers. Specifically, we aimed to investigate whether bupropion has similar effects to SSRIs and/or SNRIs acting to reduce negative biases in emotional processing, or has more specific effects on positive emotional or reward processing. Since bupropion increases dopamine function, we hypothesized that it would specifically increase positive emotional processing and reward sensitivity in a probabilistic instrumental learning task.

## Materials and methods

The authors assert that all procedures contributing to this work comply with the ethical standards of the relevant national and institutional committees on human experimentation and with the Helsinki Declaration of 1975, as revised in 2008. All participants provided written informed consent.

### Participant recruitment, screening, and randomization

A reverse power calculation using the effect sizes observed in preceding studies of other antidepressants [e.g., (8, 9)] indicated a sample size of 20 participants per treatment group would be sufficient to detect a significant difference between the two treatment groups with a power of 0.95. Therefore, a total of 40 healthy participants were recruited and deemed to be free from either current or past history of any Axis 1 DSM-IV psychiatric illness via assessment with the Structured Clinical Interview (SCID) for DSM-IV (21). They also had no physical medical conditions, were free of any medications or drugs that could impact upon the safety or effect of bupropion for at least 3 weeks and naive to the behavioral tasks.

Participants were randomly allocated to double-blind intervention with either an acute dose (150 mg) of sustained release bupropion or placebo. Administration of the treatment in identical capsules by an independent member of staff ensured that both the participant and investigator remained blind to the treatment received. Participants were stratified for gender and matched for age and National Adult Reading Test (NART)-derived verbal IQ (22). Note that an additional group of 20 participants were also recruited and randomized to a no treatment group to assess the influence of the placebo effect, the results of which are reported in Huneke et al. (23); however, all hypotheses for both studies were made a priori.

A 3 h wait period followed treatment administration since this is the t_max_ of the sustained release formulation of bupropion and allowed for testing at maximum plasma concentration (24). Participants then completed the Emotional Test Battery (ETB) and a probabilistic instrumental learning task to assess emotional and reward processing. Subjective mood was also assessed via completion of a variety of questionnaires before and after treatment administration and behavioral assessment. Firstly, the Hamilton Rating Scale for Depression (HAM-D) (25) was administered via a semi-structured interview with a trained experimenter. The rest of the questionnaires were self-report questionnaires completed on a computer and included the Adult Eysenck Personality Questionnaire (EPQ) (26), the Full Mood and Anxiety Symptom Questionnaire (MASQ), the Positive and Negative Affect Schedule (PANAS) (27), the Befindlichkeits Scale (BFS) (28), the Snaith-Hamilton Pleasure Scale (SHAPS) (29), and a side-effects questionnaire listing the side-effects most common for bupropion. The SHAPS comprises 14 items with each item describing a pleasurable situation covering one of four domains of pleasure: interests / pastimes, social interaction, sensory experience and food/drink, with a higher score indicating higher anhedonia. After treatment administration and behavioral assessment, participants repeated the PANAS, BFS, SHAPS, and side-effects questionnaires.

### Emotional test battery

The ETB (P1vital, Oxford, UK) is designed to assess the processing of a variety of affectively valenced stimuli and comprises five validated, computerized cognitive tasks named as follows: Facial Expression Recognition Task (FERT), Emotional Categorization Task (ECAT), Facial Dot-Probe Task (FDOT), Emotional Recall Task (EREC), and Emotional Recognition Memory Task (EMEM). These tasks have previously been described in full (11, 30). “In brief, the FERT comprises a series of facial expressions associated with six basic emotions: anger, disgust, fear, happy, sad and surprise at a range of different intensity levels and participants are required to identify the emotion of the face. Signal detection theory is used to provide estimates of target sensitivity (d') and beta. The ECAT comprises a series of positively and negatively valenced self-referent words and participants are required to indicate whether they would like or dislike to be referred to as each word. In the FDOT, the attentional vigilance to happy or fearful faces can be determined from participants' response latency to indicate the alignment of a dot probe appearing in the place of one of the faces. The EREC is a surprise free recall task during which participants are required to remember as many of the positively and negatively valenced self-referent words from the ECAT as they can in 2 min. Finally, the EMEM comprises self-referent words from the ECAT and previously unseen self-referent words that participants are required to classify as familiar or novel” (30). Further details for each task are provided in the Supplementary Material.

### Probabilistic instrumental learning task

The probabilistic instrumental learning task was a modified version of that described in Pessiglione et al. (19) and has previously been described in full (30). “Task stimuli consisted of two pairs of symbols with one pair associated with win outcomes (win £1 or no change) and the other associated with loss outcomes (lose £1 or no change). Each symbol in the pair corresponded to reciprocal probabilities (0.7 or 0.3) of the associated outcomes occurring.

Participants first performed a shortened, 10 trial familiarization version of the task. Participants then performed two 60 trial runs (30 win trials and 30 loss trials) with each run containing a different set of 4 symbols. Participants began the task with £5. On each trial, participants were randomly presented with a pair of symbols on a display screen for 4,000 ms, with each symbol randomly positioned either to the left or the right of a central fixation cross. Participants were required to choose between the two symbols in order to maximize their winnings. Once a choice was made, outcome feedback was provided. Participants should use the outcome feedback to gradually learn the symbol-outcome associations over time, such that they consistently choose the symbol with the high-probability win and avoid the symbol with the high-probability loss. Outcome measures were end total, amount won and amount lost, choice frequency and reaction time averaged across the two runs.”

### Statistics

Reaction times for all tasks (with the exception of the EREC where a 2 min time limit is imposed) were trimmed at the participant level: reaction times above 3 standard deviations from the mean or below 200 ms were excluded prior to calculating the mean. Data for all tasks was normally distributed allowing the use of parametric statistical tests.

Data from each task of the ETB was analyzed using a repeated measures analysis of variance (ANOVA) with treatment group (bupropion, placebo) as the between-subject factor and different within-subject factors depending on the task (FERT: face emotion; ECAT/EREC/EMEM: word valence; FDOT: face emotion, masking). Significant interactions were followed up with independent samples *t*-tests between the two treatment groups. Since previous studies have found both citalopram and reboxetine to increase the perception of ambiguous faces as happy in both healthy volunteers (8–10) and MDD patients (11), a planned comparison of the recognition of happy faces between groups was completed for the FERT.

For the probabilistic instrumental learning task, participants totaling less than the initial £5 were assumed to not have understood the task and were excluded (6 in total: 3 from the bupropion group and 3 from the placebo group). Data was then averaged across the two runs and analyzed using independent samples *t*-tests between the two treatment groups.

## Results

### Participant demographics and characterization

There were no significant differences between treatment groups with regards to gender, age, NART-derived verbal IQ and baseline scores on the HAM-D and self-report questionnaires (Table S1).

### Changes in subjective mood

There were no significant main effects of treatment group or time by treatment group interactions for any of the questionnaires measuring subjective mood, apart from the SHAPS. A time by treatment group interaction was observed for the SHAPS [*F*_(138)_ = 5.95, *p* < 0.05] with a significant difference in the change in SHAPS score over time between the placebo and bupropion groups [*t*_(38)_ = −2.44, *p* < 0.05]. Paired *t*-tests found SHAPS score to decrease in the placebo group, although not significantly (−1.25 ± 3.77, *p* = 0.15), but increase in the bupropion group with a trend toward significance (+1.40 ± 3.17, *p* = 0.06). Side-effect ratings were very low with the majority of participants rating that side-effects were absent (1.00) pre- and post-treatment (Table S2).

### Acute effects of bupropion on emotional processing

#### Facial expression recognition task

**Figure 1:**
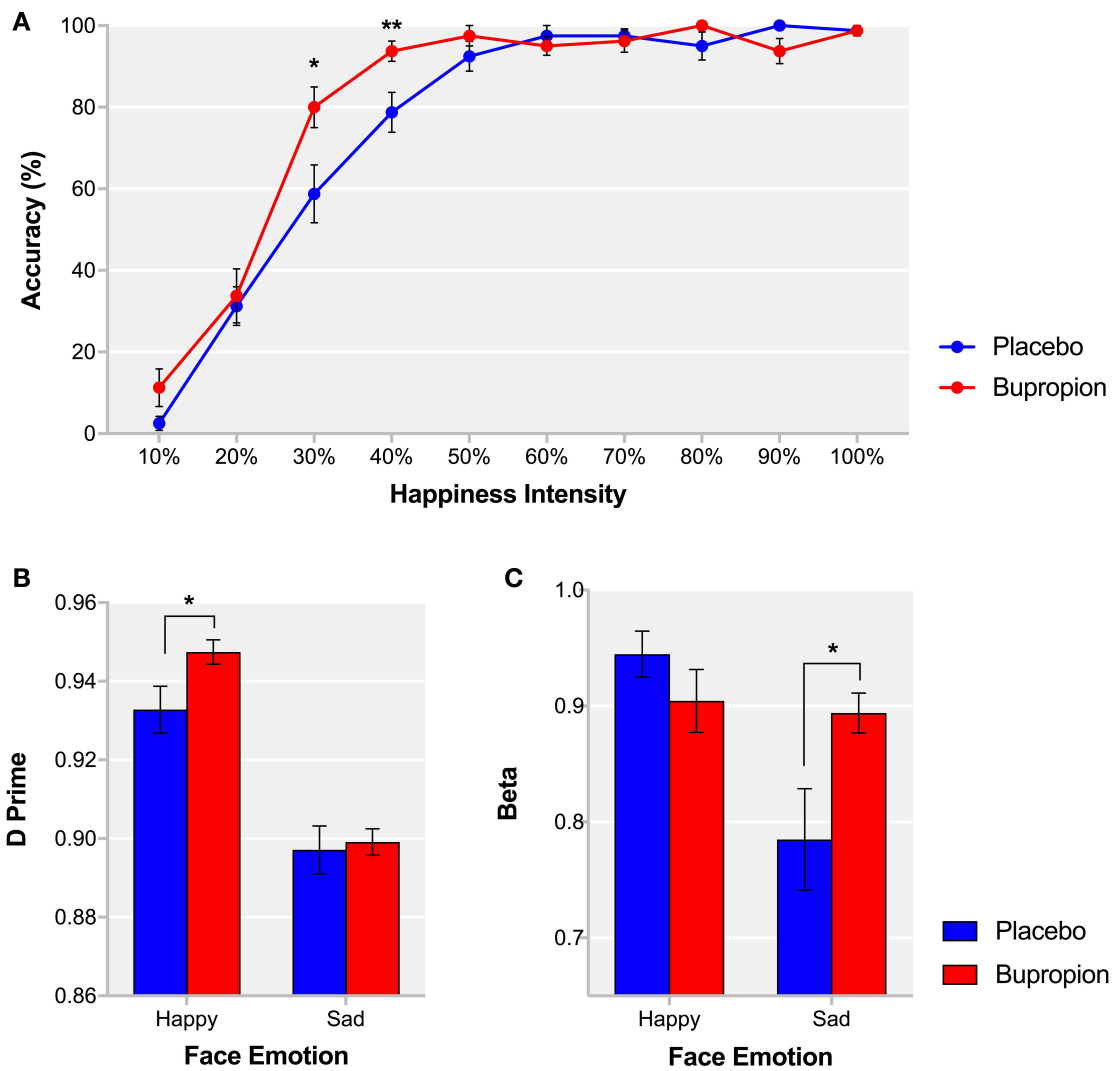
FERT **(A)** % accuracy for each happiness intensity and signal detection derived **(B)** d' and **(C)** beta for happy and sad faces for each treatment group. Values are reported as means ± SEM. Asterisks denote the degree of significance obtained for planned comparisons (*p < 0.05, **p < 0.01).

During the FERT, participants are required to recognize emotional facial expressions. Signal detection theory is used to provide estimates of target sensitivity (d') and beta. For % accuracy in recognizing emotional facial expressions, there was no significant main effect of treatment group [*F*_(1, 38)_ = 0.97, *p* = 0.33] or face emotion by treatment group interaction [*F*_(5, 190)_ = 0.89, *p* = 0.49]. Correspondingly, there was also no significant main effect of treatment group [*F*_(1, 38)_ = 1.00, *p* = 0.32] or face emotion by treatment group interaction [*F*_(5, 190)_ = 0.43, *p* = 0.83] for d'. In a planned comparison of the recognition of happy faces between groups, the bupropion group were found to show significantly higher % accuracy [*t*_(38)_ = −2.33, *p* < 0.05] and d' [*t*_(38)_ = −2.18, *p* < 0.05] for happy faces than the placebo group (Figure 1B). Furthermore, a significant intensity of face emotion by treatment group interaction was found for the % accuracy for recognizing happy faces [*F*_(9, 342)_ = 3.14, *p* < 0.01], with the bupropion group displaying significantly higher % accuracy for recognizing happy faces at lower intensities than the placebo group [30% happiness intensity: *t*_(38)_ = −2.45, *p* < 0.05; 40% happiness intensity: *t*_(38)_ = −2.73, *p* < 0.01] (Figure 1A). There was a trend toward significance for a face emotion by treatment group interaction for beta [*F*_(5, 185)_ = 2.17, *p* = 0.06] with independent *t*-tests finding an effect of treatment group on the beta for sad faces only. The bupropion group displayed a significantly higher beta value for sad faces compared to the placebo group [*t*_(38)_ = −2.32, *p* < 0.05], indicating bupropion may induce a response bias away from sad faces (Figure 1C).

There was no significant main effect of treatment group [*F*_(1, 37)_ = 0.01, *p* = 0.94] or face emotion by treatment group interaction [*F*_(5, 185)_ = 0.35, *p* = 0.88] for reaction time.

#### Emotional categorization task

During the ECAT, participants are required indicate as quickly as they can whether they would like or dislike to be referred to as various positively and negatively valenced words. There was no significant main effect of treatment group [*F*_(1, 38)_ = 3.11, *p* = 0.09] or word valence by treatment group interaction [*F*_(1, 38)_ = 0.01, *p* = 0.91] for reaction time.

#### Facial dot-probe task

**Figure 2:**
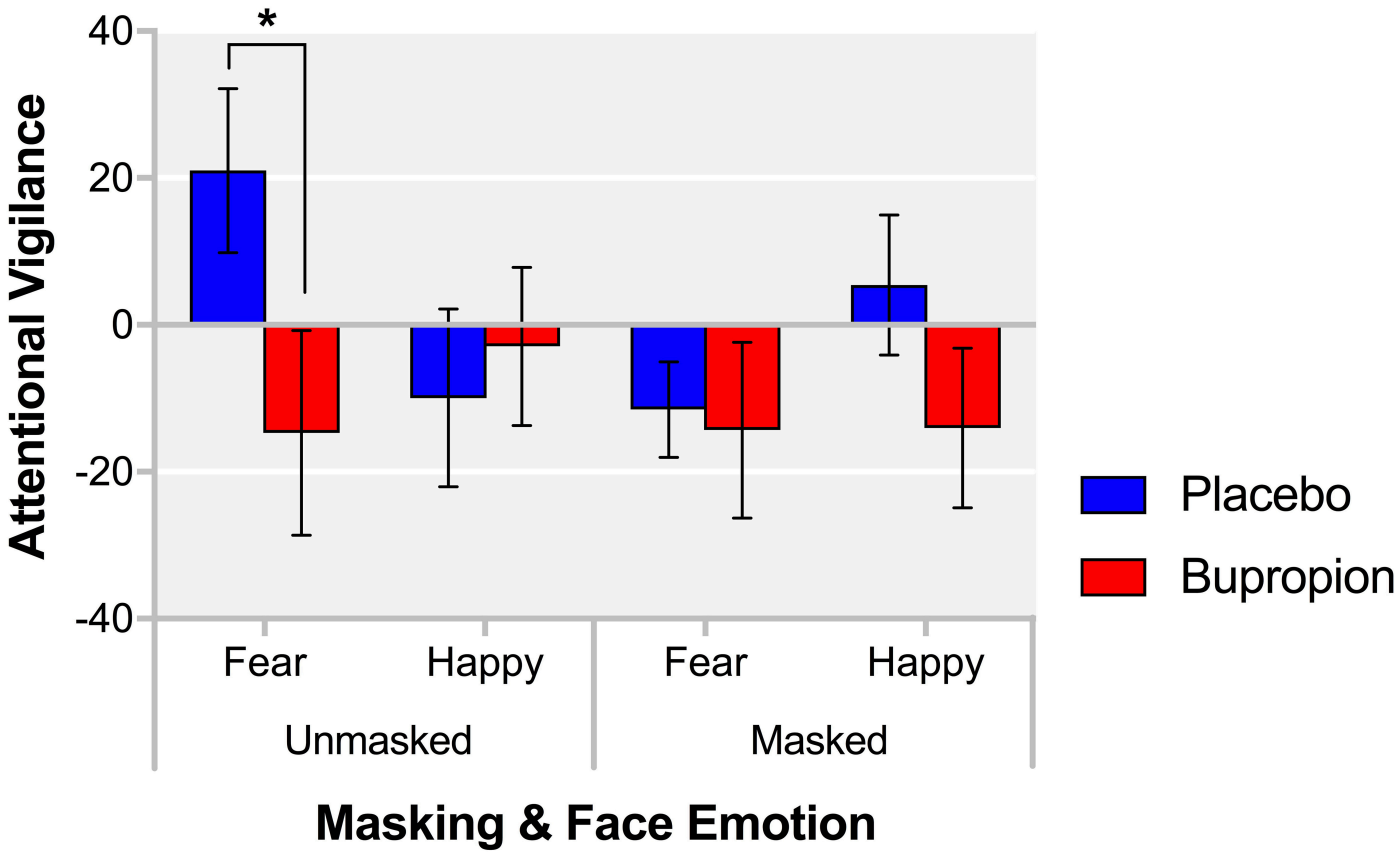
FDOT attentional vigilance for each masking and face emotion condition for each treatment group. Values are reported as means ± SEM. Asterisks denote the degree of significance obtained for planned comparisons (*p < 0.05).

In the FDOT, the attentional vigilance to happy or fearful faces can be determined from participants' response latency to indicate the alignment of a dot probe appearing in the place of one of the faces. There was a significant face emotion by masking by treatment group interaction for attentional vigilance [*F*_(1, 38)_ = 5.45, *p* < 0.05]. This was found to be driven by a significant face emotion by treatment group interaction for unmasked faces [*F*_(1, 38)_ = 4.30, *p* < 0.05], with the bupropion group displaying significantly reduced explicit attentional vigilance for unmasked fearful faces compared to the placebo group [*t*_(38)_ = 2.00, *p* < 0.05] (Figure 2).

#### Emotional recall task

The EREC is a surprise free recall task during which participants are required to remember as many of the positively and negatively valenced self-referent words from the ECAT as they can in 2 min. There was no significant main effect of treatment group or word valence by treatment group interaction for both number of words correctly [*F*_(1, 38)_ = 1.22, *p* = 0.28; *F*_(1, 38)_ = 2.00, *p* = 0.17] and falsely [*F*_(1, 38)_ = 0.17, *p* = 0.68; *F*_(1, 38)_ = 0.38, *p* = 0.54] recalled.

#### Emotional recognition memory task

**Figure 3:**
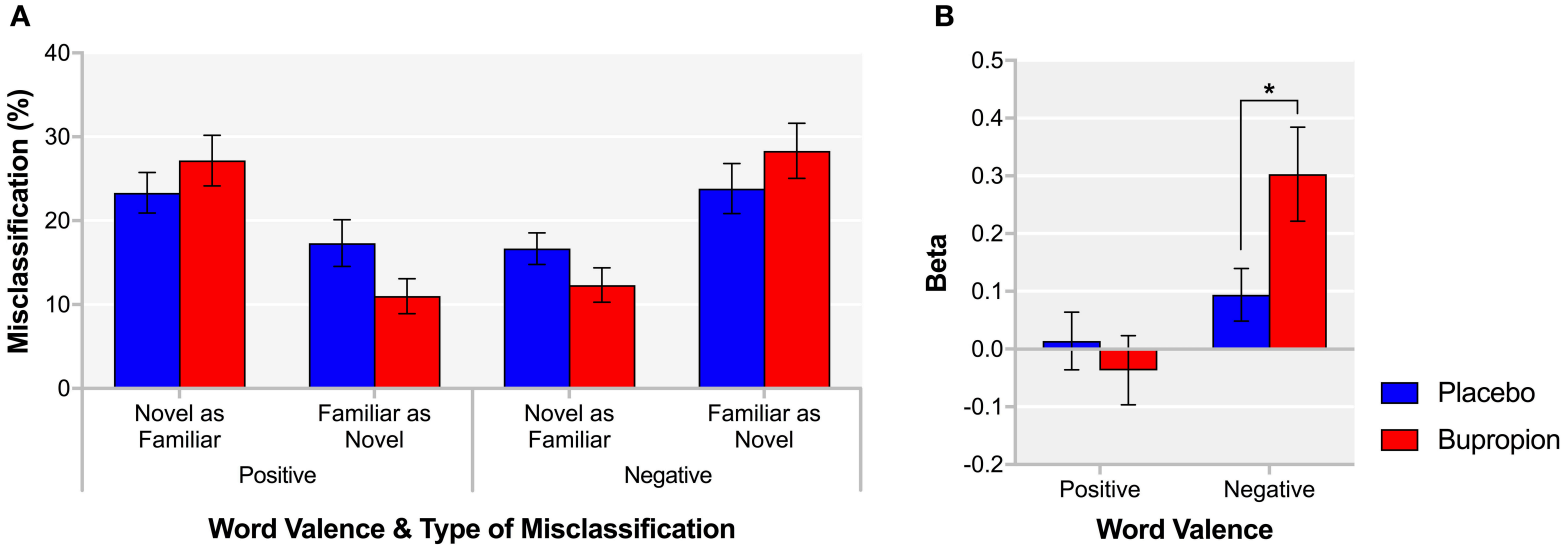
EMEM **(A)** % misclassification and **(B)** beta for each word valence and treatment group. Values are reported as means ± SEM. Asterisks denote the degree of significance obtained for planned comparisons (*p < 0.05).

The EMEM comprises the words from the ECAT and previously unseen words that participants are required to classify as familiar or novel. A significant word valence by treatment group interaction was found for both novel words misclassified as familiar [*F*_(1, 38)_ = 10.24, *p* < 0.01] and familiar words misclassified as novel [*F*_(1, 38)_ = 7.34, *p* < 0.01]. Figure 3A suggests that bupropion increases the familiarity of positive words and decreases the familiarity of negative words. When considering just false alarms (novel words misclassified as familiar), there was no significant difference between groups for positive words [*t*_(38)_ = 0.65, *p* = 0.52] but the bupropion group displayed significantly increased beta for negative words compared to the placebo group [*t*_(38)_ = −2.25, *p* < 0.05] (Figure 3B).

#### Acute effects of bupropion on reward processing

**Figure 4:**
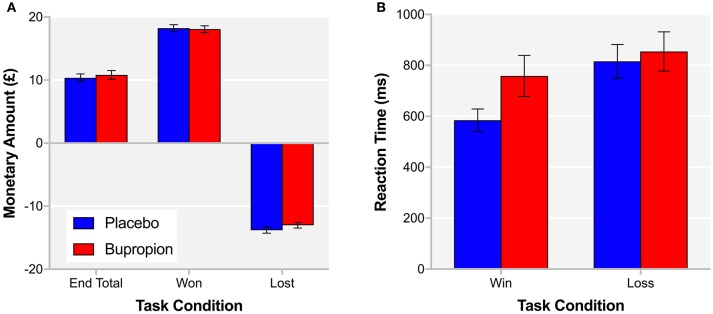
**(A)** End total, amount won and amount lost and **(B)** reaction time for the win and loss conditions of the probabilistic instrumental learning task for each treatment group. Values are reported as means ± SEM.

Independent samples *t*-tests did not find a significant difference between treatment groups for the total monetary amount at the end of the task [*t*_(38)_ = −0.51, *p* = 0.61], the amount won [*t*_(38_ = 0.20, *p* = 0.85] or the amount lost [*t*_(38)_ = −1.18, *p* = 0.24] (Figure 4A). A repeated measures ANOVA did find a task condition by treatment group interaction for reaction time [*F*_(1, 38)_ = 5.73, *p* < 0.05], with the bupropion group displaying slower reaction times in the win vs. loss condition compared to the placebo group (Figure 4B).

**Figure 5:**
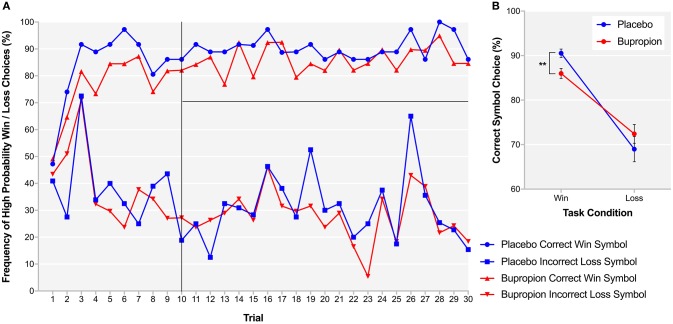
**(A)** Learning curves for each treatment group depicting trial-by-trial the proportion of participants that chose the correct symbol in the win condition, associated with high-probability win (top lines) and the incorrect symbol in the loss condition, associated with high-probability loss (bottom lines) during the probabilistic instrumental learning task. **(B)** Proportion of participants choosing the correct symbol in the win and loss conditions averaged over the last 20 trials of the probabilistic instrumental learning task where learning had plateaued. Asterisks denote the degree of significance obtained for planned comparisons (**p < 0.01).

In order to provide more temporal information about reward learning differences between treatment groups, learning curves were produced for each treatment group depicting trial-by-trial the proportion of participants that chose the correct symbol in the win condition, associated with high-probability win and the incorrect symbol in the loss condition, associated with high-probability loss (Figure 5A). Both treatment groups learnt to choose the high-probability win and avoid the high-probability loss by about trial 10. To assess reward sensitivity after learning, the proportion of participants choosing the correct symbol in the win and loss conditions was averaged over the remaining 20 trials of the task where learning had plateaued (31). The bupropion group was found to be significantly less likely to choose the correct symbol in the win condition compared to placebo [*t*_(38)_ = 3.00, *p* < 0.01] (Figure 5B).

## Discussion

The present study aimed to investigate whether bupropion has similar effects to SSRIs and/or SNRIs acting to reduce negative biases in emotional processing, or has more specific effects on positive emotional or reward processing. Since bupropion increased dopamine function, we hypothesized that it would specifically increase positive emotional processing and reward sensitivity on a probabilistic instrumental learning task similarly to other dopamine acting drugs (19). An acute dose of bupropion significantly increased the recognition of ambiguous faces as happy, decreased response bias toward sad faces and reduced attentional vigilance for fearful faces compared to placebo. Bupropion also reduced negative bias compared to placebo in the (EMEM). There was no evidence that bupropion enhanced reward processing or learning; rather the drug treatment was associated with reduced sensitivity to high-probability wins and increase in score on a subjective measure of anhedonia compared to placebo.

### Emotional processing

Whilst an acute dose of bupropion did produce a slight increase in positive emotional processing, with an increase in the recognition of ambiguous faces as happy, it was actually found to have stronger effects on decreasing negative emotional processing, with a decrease in the response bias for sad faces, attentional vigilance to fearful faces and negative bias in emotional recognition compared to placebo. These effects on emotional processing are similar to those seen with SSRIs and/or SNRIs (8–10) and have been hypothesized to be an early mechanism of antidepressant drug action; by reversing negative biases in depression and reducing the influence of this maintaining factor (4–7).

The profile of effects overlaps with the effects of SNRIs to a greater extent than SSRIs (6). Specifically, in addition to the positive biasing effect, SSRIs paradoxically increase fear processing early in treatment. For example, an acute dose of the SSRI citalopram was found to increase the startle response (32) and the recognition of fearful faces (33). However, an acute dose of the SNRI reboxetine was not found to have any effect on fear processing (9), similarly to bupropion in the present study. Reboxetine has also been found to increase the recognition of happy faces in the FERT and alter the balance of memory for self-referent words, causing an increase in recall of positive words or decrease in the recall of negative words (9–11). Whilst reboxetine acts primarily as an SNRI, some have reported that it also increases dopaminergic activity in the frontal cortex (34, 35). Likewise, although dopamine reuptake inhibition is the mechanism of action most commonly attributed to bupropion, the exact neuropharmacological actions of bupropion remain elusive, due to different actions *in vitro* vs. *in vivo* (36, 37). *In vitro*, bupropion is more potent at inhibiting dopamine than noradrenaline reuptake (IC_50_ of 2.0 and 5.0, respectively) (36) but the inhibition of dopamine reuptake itself is not particularly robust and was not thought to have pharmacological relevance (38). In contrast, *in vivo*, an acute dose of bupropion has been found to affect the firing rate of noradrenaline neurons in the locus coeruleus of the rat at doses more similar to those required for antidepressant-like activity in animal models (39, 40). It seems that the effects of bupropion on emotional processing may be mediated via noradrenaline and/or dopamine and further research is required in this area.

### Reward processing

It has previously been shown that administration of drugs with dopaminergic enhancing activity can improve performance on probabilistic instrumental learning tasks in healthy volunteers. For example, administration of L-DOPA, the metabolic precursor of dopamine, was found to significantly increase the likelihood of choosing the stimulus associated with high-probability win and subsequently the amount of money won during a probabilistic instrumental learning task, compared to the dopamine receptor antagonist haloperidol (19). Therefore, it could be expected that an acute dose of bupropion with dopaminergic enhancing activity would also improve performance on a probabilistic instrumental learning task in healthy volunteers; however, this was not found to be the case. Instead, bupropion reduced the likelihood of choosing the stimulus associated with high-probability win. Such a profile is similar to that seen in depression itself (16–18) and bupropion may therefore be predicted to worsen anhedonia at least early in treatment. However, care must be taken when interpreting these results obtained in a sample of healthy volunteers with regards to depression. Key differences in reward and emotional processing between healthy and depressed individuals are likely to have a large impact upon the effects of bupropion.

Indeed, in a healthy system with roof levels of dopamine, acute inhibition of the reuptake of dopamine could lead to a paradoxical decrease in cell firing via activation of the presynaptic autoreceptors (41). It has previously been shown, at least in rats, than an acute dose of bupropion induced an autoreceptor-mediated reduction in the firing of brain stem dopamine neurons (40, 42). Subsequent down-regulation of the autoreceptors may be required to reverse these effects, allow an increase in the levels of dopamine in the synapse and improve reward processing in healthy participants (43).

Bupropion could also differentially affect the phasic vs. tonic firing of dopamine neurons. Phasic firing refers to a transient burst of firing following presynaptic input in response to a stimulus and plays a crucial role in associative reward learning (44). Tonic firing refers to sustained firing at a constant frequency regulated by frontal activity in order to set the background level of dopamine and subsequently the responsivity of the dopaminergic system (44). Administration of bupropion may act to increase tonic levels of dopamine but as a result decrease the responsivity of the dopaminergic system such that phasic firing is actually reduced. This may reduce reward discriminability such that the participant believes the neutral and win outcomes are of a similar magnitude (45). As such participants fail to or are slower to learn the association of a particular stimulus with high-probability win, thereby disrupting instrumental reward learning.

SSRIs have also been shown to reduce reward processing, for example, short-term treatment with the SSRI citalopram, but not the SNRI reboxetine, reduced ventral striatal, and ventral medial/orbitofrontal cortex activation in response to chocolate reward (13). However, more recent research suggests that longer-term treatment with SSRIs has a beneficial effect on reward processing, with 2 week citalopram treatment increasing reward learning and the effort applied to obtain rewards (46). Similarly, chronic administration of bupropion may be required for the beneficial effects on reward processing, in correspondence with the delay in the action of antidepressants to produce a clinical important therapeutic effect. Further research into the longer-term effects of bupropion on reward processing in MDD patients is required.

The bupropion group also displayed a slight increase in SHAPS score, and therefore, anhedonia, compared to placebo over time. The slight increase in anhedonia may be associated with acute adverse effects of bupropion on reward processing and may have clinical implications when starting treatment with bupropion. With the exception of the SHAPS, all of these effects occurred in the absence of any changes in subjective mood. This provides evidence that antidepressants acting on a range of neurotransmitters, including serotonin, noradrenaline and dopamine, all have early effects on the processing of affective stimuli prior to mood improvement. Our results therefore further support the neuropsychological theory of antidepressant action.

## Conclusion

Despite its alternative mechanism of action involving dopamine, an acute dose of bupropion appears to have a similar profile of effects on emotional and reward processing to other antidepressants. Acute bupropion acts to restore the balance between negative and positive emotional processing but with adverse effects on reward processing and anhedonia, at least in healthy participants. The beneficial effects of bupropion on reward processing may only occur in MDD individuals or following repeated administration. As such, there is a dissociation of the acute effects of bupropion on positive emotional processing and reward processing in healthy volunteers indicating they may be different processes in the manifestation of the symptom clusters in MDD; however, the roles of different neurotransmitters, how they interact and their downstream effects needs to be unraveled. If the adverse effects of acute bupropion on reward processing are found to occur in MDD individuals, the use of bupropion to specifically target anhedonia should be monitored early in treatment for any initial worsening of anhedonic symptoms.

## Ethics statement

This study was carried out in accordance with the recommendations of the Central University Research Ethics Committee (CUREC, University of Oxford) with written informed consent from all subjects. All subjects gave written informed consent in accordance with the Declaration of Helsinki. The protocol was approved by the Central University Research Ethics Committee.

## Author contributions

AW, RB, and NH recruited volunteers for this study and analyzed the data; CH, MB, and PC were involved in study and task design and oversaw the running and analysis of the study. AW drafted the first draft of the paper and all authors revised this draft.

### Conflict of interest statement

The authors declare that the research was conducted in the absence of any commercial or financial relationships that could be construed as a potential conflict of interest.
