# Supplementary material for: A Dissociation of the Acute Effects of Bupropion on Positive Emotional Processing and Reward Processing in Healthy Volunteers
Source: Front Psychiatry. 2018 Oct 16;9:482. doi: 10.3389/fpsyt.2018.00482 (PMC6198095; doi:10.3389/fpsyt.2018.00482)

**Supplementary Material**

*Facial Expression Recognition Task (FERT).* Task stimuli consisted of a series of facial expressions associated with six basic emotions: anger, disgust, fear, happy, sad, surprise and neutral. Each emotion also had a range of different intensity levels by using faces morphed between neutral (0%) and full intensity (100%) in 10% steps (Young et al., 1997). For each emotion, there were 4 faces for each intensity level, totalling 280 emotional facial expressions randomly presented in the centre of a display screen, each for 500 ms, divided across 4 blocks. Participants were instructed to indicate the emotional expression on the face by pressing a correspondingly labelled button. Outcome measures were percentage accuracy for facial expression recognition, percentage misclassifications and average reaction time for correct responses for each emotion. Signal detection theory was also used to provide estimates of target sensitivity (d’) and beta. Increased beta indicates decrease response bias for a particular emotional facial expression. To calculate d’ and beta, percentage accuracy and percentage misclassification were first divided by 100 then the formulae used were as follows, where x is the percentage accuracy/100 and y is the percentage misclassification/100:

d’ = 0.5+((x-y)*(1+x-y)/((4*x)*(1-y)))

beta = (x*(1-x)-y*(1-y))/(x*(1-x)+y*(1-y))

*Emotional Categorisation Task (ECAT).* A series of 60 words, selected to be either positive or negative descriptors of personality, were randomly presented in the centre of a display screen, each for 500 ms. The personality descriptive words were matched for word length and ratings of frequency and meaningfulness. Participants were instructed to indicate whether they would like or dislike to be referred to as each personality descriptive word by pressing a correspondingly labelled button. The outcome measure was average reaction time for correct responses for positive versus negative self-referent personality descriptive words.

*Facial Dot-Probe Task (FDOT).* During each trial of the FDOT, a pair of faces was presented vertically on a display screen prior to the appearance of a dot-probe. There were 96 trials in total, with 48 trials showing a positive facial expression (happy) and 48 trials showing a negative facial expression (fearful), each time paired with a neutral facial expression. Additionally, the pairs of faces could either be unmasked and presented for 500 ms to assess explicit processing or briefly presented for 14 ms and then replaced by a jumbled face mask to assess implicit emotional processing. The pairs of faces were presented in a random order, divided across 4 blocks. The dot-probe appeared either in the same position as the emotional facial expression (congruent trials) or in the opposite position to the emotional facial expression (incongruent trials). The dot-probe comprised a pair of dots that could either be vertically or horizontally aligned and participants were instructed to indicate the alignment of the dot-probe by pressing a correspondingly labelled button as quickly as possible. Reaction time was recorded and used to calculate attentional vigilance scores for each face emotion.

Attentional vigilance = RT incongruent trials – RT congruent trials

*Emotional Recall Task (EREC).* Participants were given a surprise free recall task where they were asked to write down as many of the self-referent words that they could remember from the ECAT within a 2 minute time limit. The totals for positive versus negative self-referent words correctly and falsely recalled were calculated.

*Emotional Recognition Memory Task (EMEM).* A series of 120 words, comprising the positive and negative self-referent words included in the ECAT and previously unseen distractor self-referent words were randomly presented in the centre of a display screen, each for 500 ms. Participants were instructed to indicate whether they had previously been presented with the self-referent word or not. Outcome measures were percentage accuracy, percentage misclassification of familiar words as novel and vice versa and average reaction time for correct responses for positive versus negative self-referent words. Signal detection theory was also used to provide estimates of target sensitivity (d’) and beta / response bias for positive versus negative self-referent words.


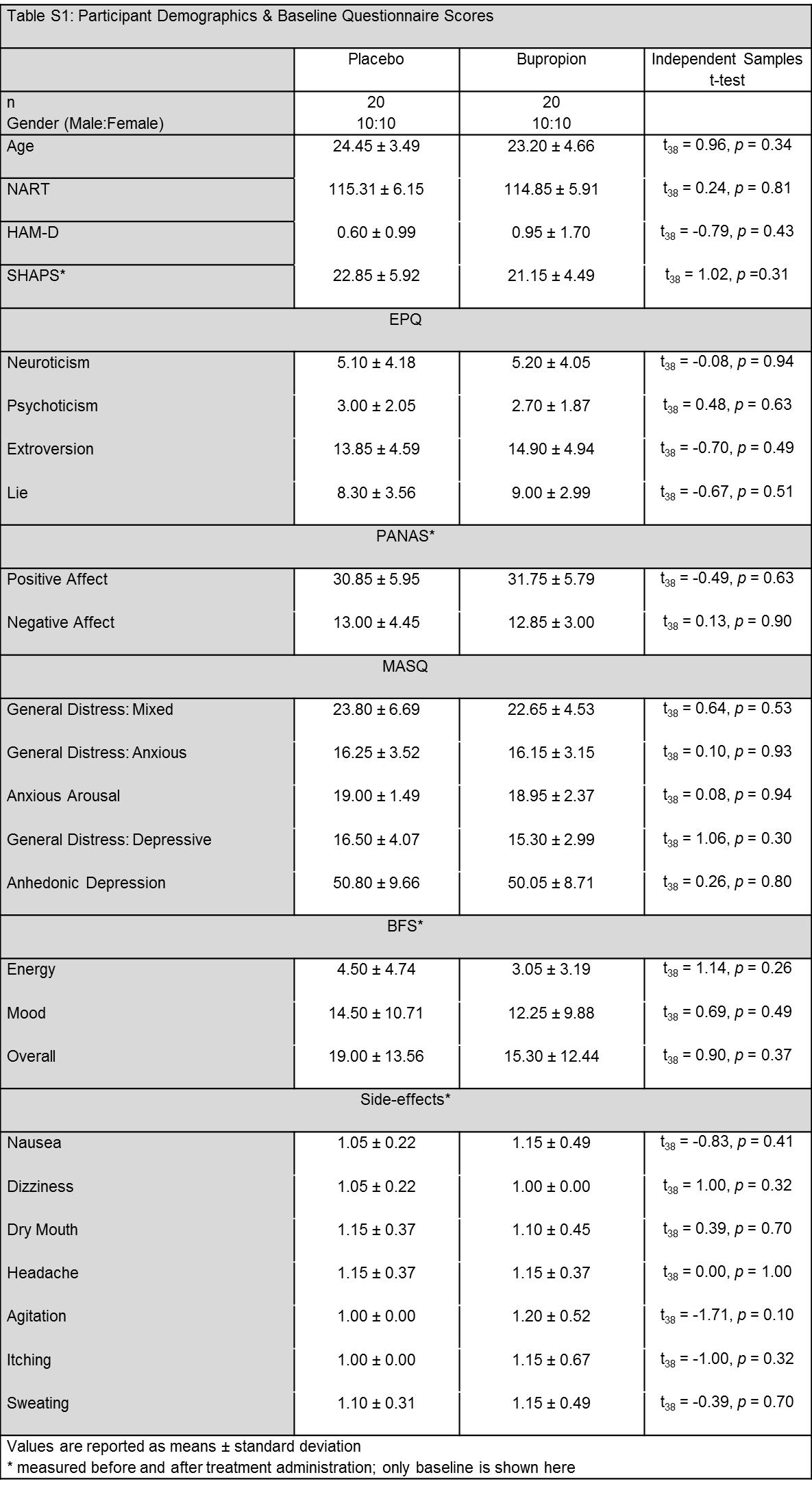


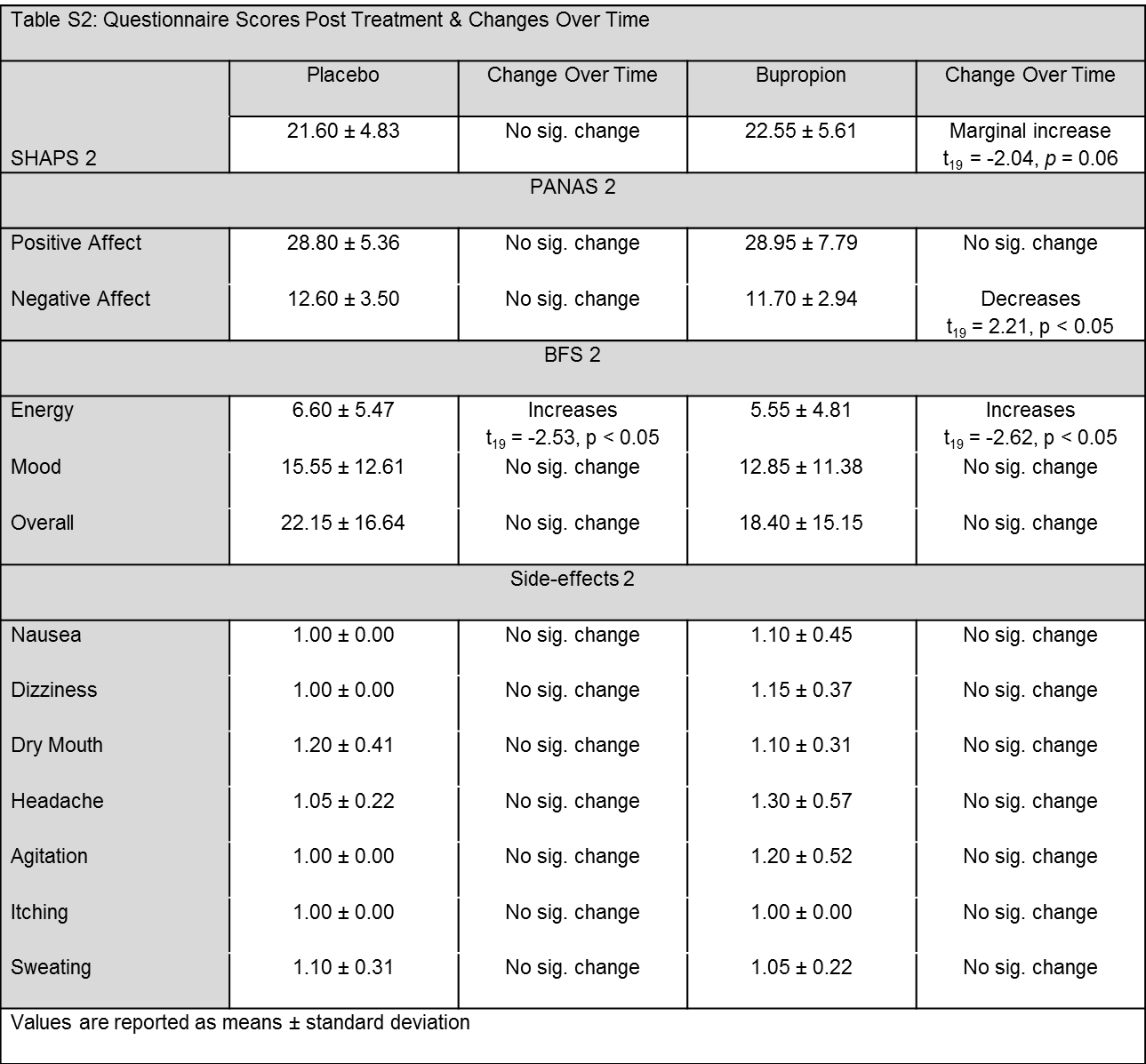

Supplement: Supplementary file 1 [file Table_1.DOCX]
